# Late Breakage of a Dual-Mobility Polyethylene Insert in a Revision Total Hip Arthroplasty: An Unusual Failure Mode

**DOI:** 10.1016/j.artd.2021.07.013

**Published:** 2021-10-14

**Authors:** Daniel Rodríguez Pérez, José Luis Agulló Ferre, Marcos Del Carmen Rodríguez, Carles Tramunt Monsonet

**Affiliations:** Department of Orthopedic Surgery, Hospital Universitario de Bellvitge, Barcelona, Spain

**Keywords:** Dual-mobility, Polyethylene, Hip, Total hip arthroplasty, Luxation, Intra-prosthetic dislocation

## Abstract

The use of dual-mobility cups has gained popularity in recent years. Thus, surgeons can expect an increase in known and new causes of failure. We report a previously undescribed form of a late intraprosthetic dislocation consisting of a complete breakage of a polyethylene mobile bearing that suffered a dislocation 3 months after its implantation. Two years later, he began feeling anterior groin pain and suffered gait changes. Computed tomography scan revealed an eccentric alignment of the mobile polyethylene bearing suggestive of poly wear. During the revision surgery, the polyethylene was found to be split in 2. Possible causes of this complication are proposed. Our case shows a previously unreported implant-specific complication, so surgeons can identify it.

## Introduction

The concept of a dual-mobility cup (DMC) was introduced in 1974 to improve total hip arthroplasty (THA) stability [1]. DMC combines a prosthetic femoral head with a large, mobile, retentive polyethylene bearing that articulates with a metal cup, which is fixed to the acetabulum. The large outer diameter of the bearing provides a jump distance that reduces the risk of dislocation. In addition, the large diameter of the mobile polyethylene bearing increases range of movement [[2], [3], [4], [5], [6]], reduces the likelihood of impingement, and increases the “safe zone” where the prosthesis components can be securely implanted [[7], [8], [9], [10]]. Since their inception, DMCs — both modular and monoblock designs — have evolved to increase their lifespan, the most relevant updates being the introduction of a hydroxyapatite coating on the cup, the redesign of the chamfer and retention mechanism of the liner, and the introduction of cross-linked polyethylene [3].

The use of DMCs has surged upward in recent years [11,12]; therefore, an increase in the number of incident failures can be expected, including some new modes of failure. To date, the described causes of DMC failure can be classified into 3 groups: femoral loosening, cup loosening, and intraprosthetic dislocation (IPD) [4,6,[13], [14], [15], [16], [17]]. IPD is a specific complication of DMC and occurs when the femoral head separates from the retentive polyethylene bearing. Some authors consider it a long-term complication related to polyethylene wear, distinguishing it from early traumatic complications [[18], [19], [20], [21]], which can occur after a closed reduction is attempted for a DMC dislocation [15,22]. Long-term IPD [5,[18], [19], [20], [21]] is a rare event (1.1% [23]) whose mean time of presentation is between 8 and 11 years after surgery, but it seems that the evolution of the implants and the selection of appropriate patients have reduced its incidence [2,3,5,15,24,25].

We present an unusual and, to our knowledge, previously unreported mode of DMC failure: a complete catastrophic cracking of the polyethylene bearing after a closed luxation reduction.

## Case history

**Figure 1:**
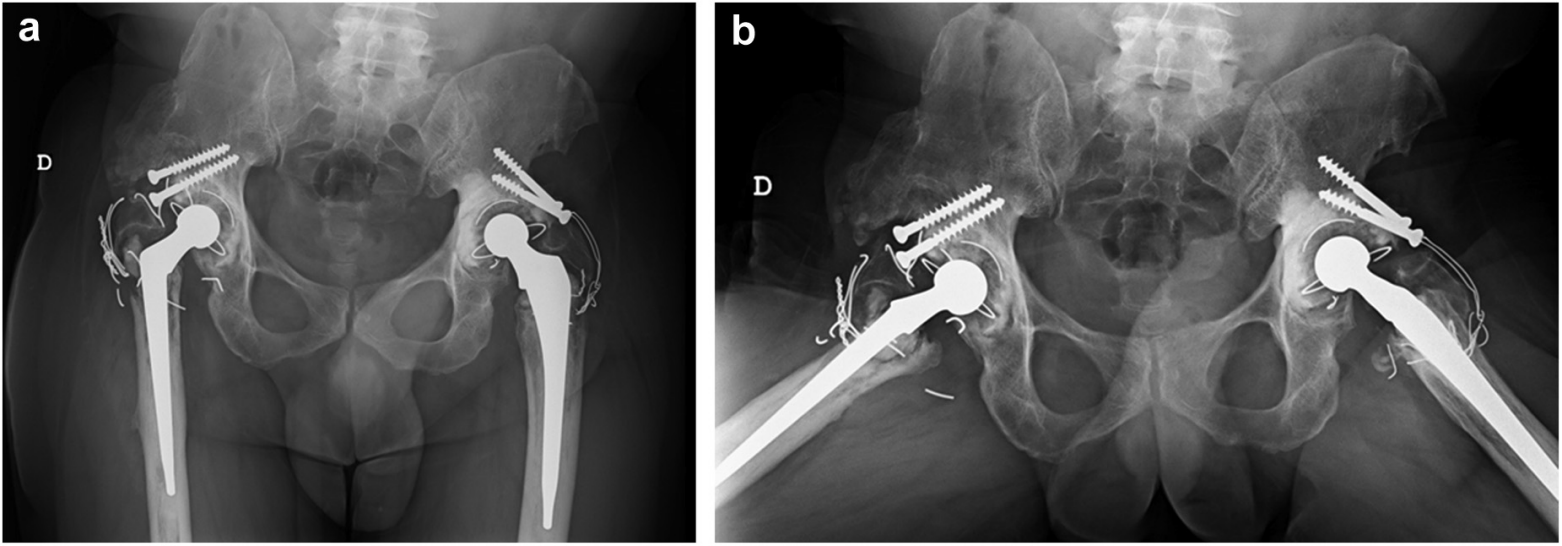
Anteroposterior (a) and frog-leg lateral (b) radiographs of the pelvis showing significant eccentric wear of the polyethylene mobile bearing and osteolysis around the right cup (DeLee and Charnley zone III) and the stem (Gruen zones 1 and 7). Some broken wires from a failed trochanteric fixation were also present.

The authors of the following case report obtained the patient's consent to having his data being submitted for publication. A 59-year-old man (body mass index: 27.5) was admitted to our hospital with progressive anterior groin pain and gait changes. He had undergone a right-side THA 16 years prior at another institution and reported an excellent functional outcome since. Radiographs demonstrated significant eccentric wear of the polyethylene bearing with osteolysis around the cup and the stem. Broken wires from a failed trochanteric fixation were also present (Fig. 1a and b). The patient, who showed no spinopelvic imbalance, was proposed as a candidate for revision surgery with a diagnosis of aseptic loosening, polyethylene wear, and pseudoarthrosis of the greater trochanter.

**Figure 2:**
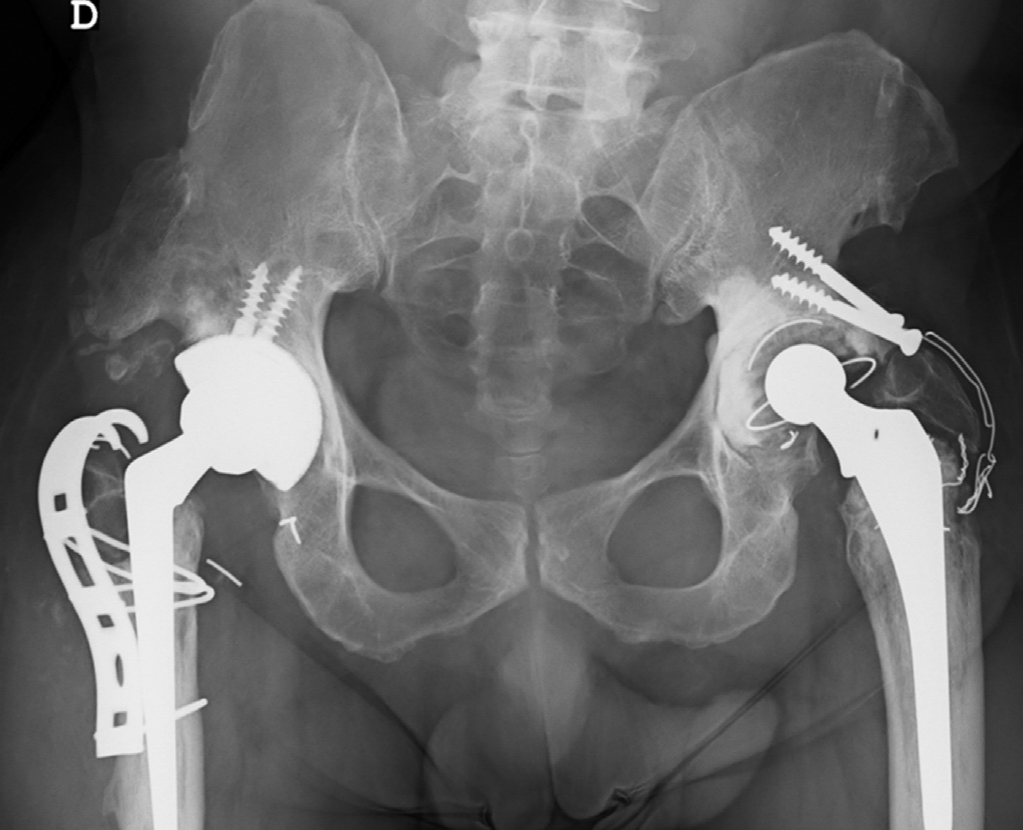
Anteroposterior radiograph of the pelvis showing the results of the revision surgery.

The same posterolateral approach was used. The loose cup was removed and replaced with a DMC construct consisting of a G7 OsseoTi multihole cup (Zimmer-Biomet, Warsaw, IN), a dual-mobility liner, an E1-infused dual-mobility highly crosslinked polyethylene bearing, and a 28-mm Protasul S-30 head (Zimmer-Biomet, Warsaw, IN). The broken wires were removed, and the greater trochanter was stabilized by means of a BMP cobalt chrome trochanteric grip plate (Zimmer-Biomet, Warsaw, IN) (Fig. 2). The stem was stable; therefore, it was not replaced. The patient was discharged 7 days after surgery and was allowed to ambulate with partial weight-bearing for 2 weeks and full weight-bearing thereafter. There were no postoperative complications, and intraoperative cultures were negative.

**Figure 3:**
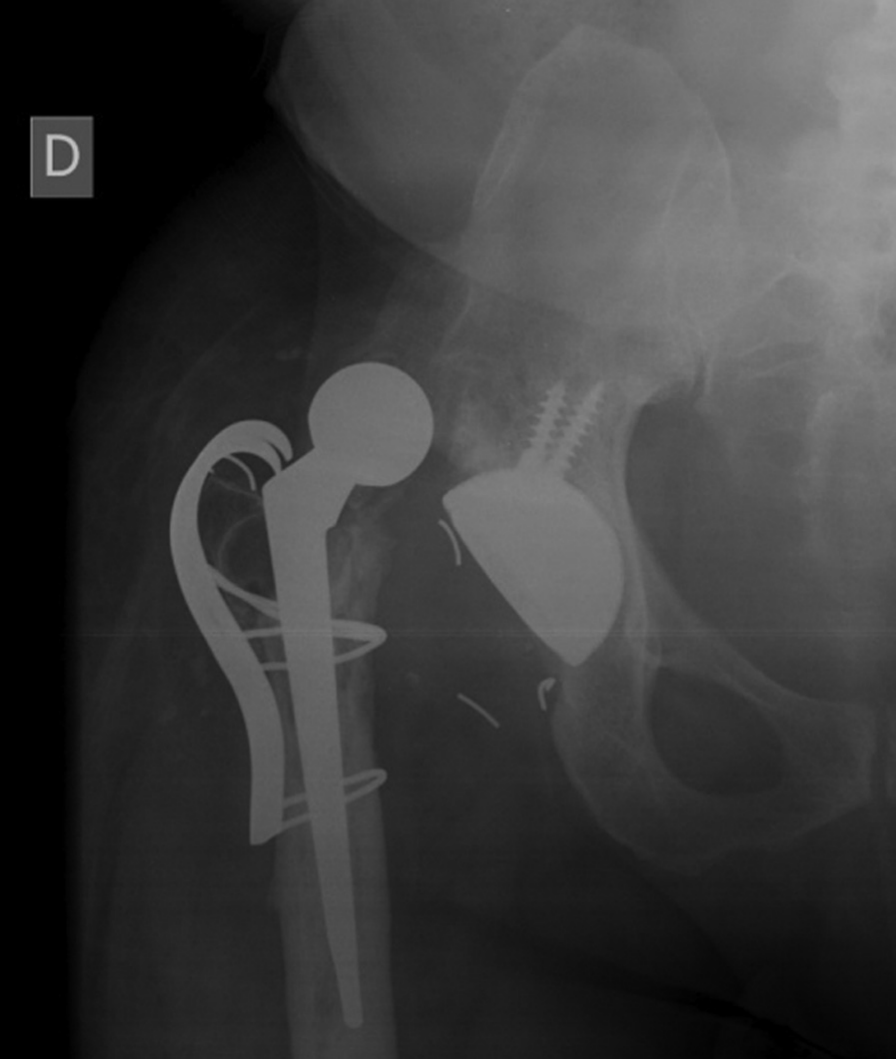
Anteroposterior hip radiograph showing prosthetic hip dislocation.

Three months later, the patient was admitted to the emergency department with superior-posterior prosthetic hip dislocation after a fall (Fig. 3). Closed reduction was performed under sedation and image intensifier control [18]. The radiograph showed concentric head positioning and no impingement between the trochanteric plate and the cup in the entire range of movement.

**Figure 4:**
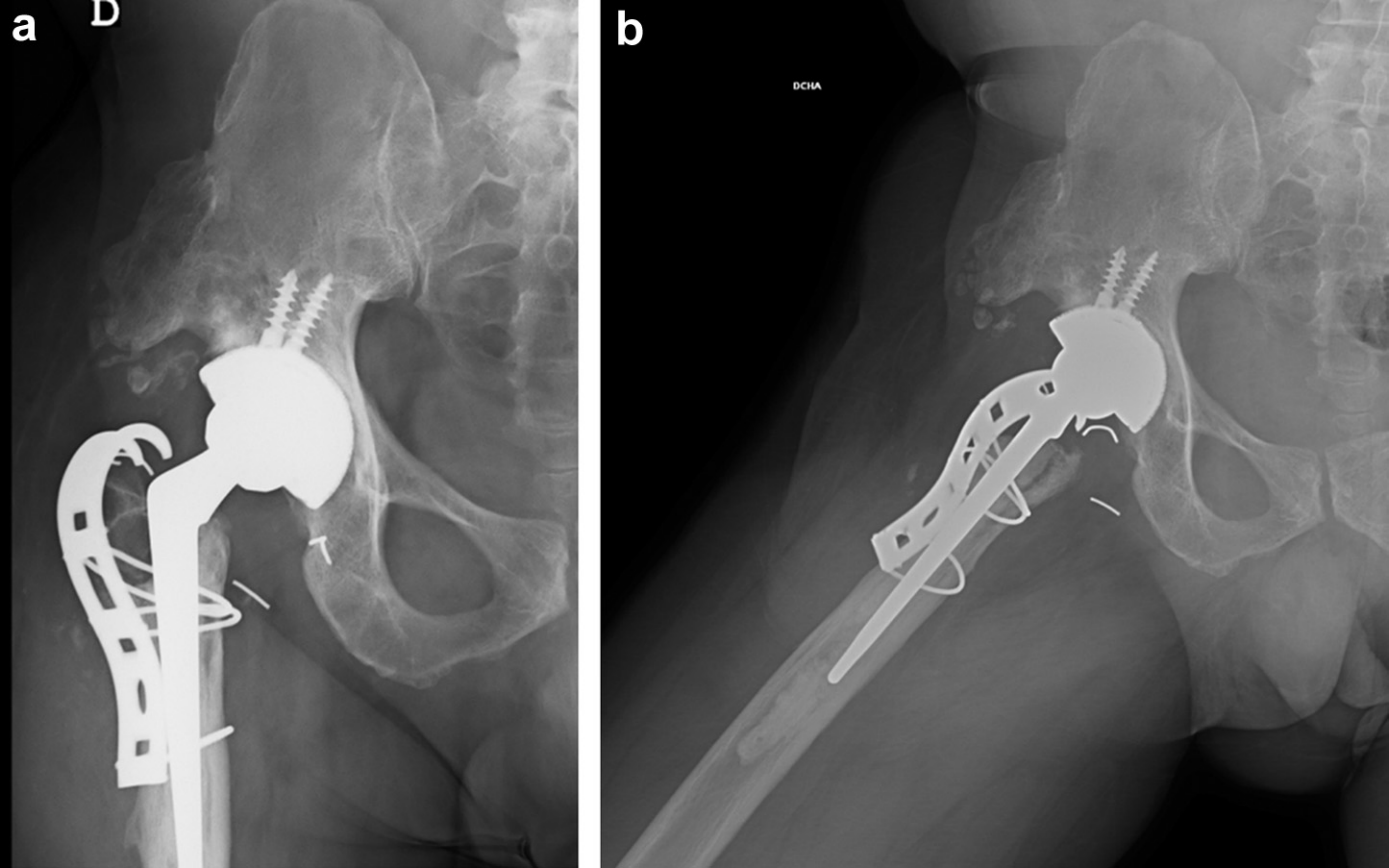
Anteroposterior (a) and frog-leg lateral (b) radiographies of the pelvis demonstrating good concentric reduction of the dislocation.

**Figure 5:**
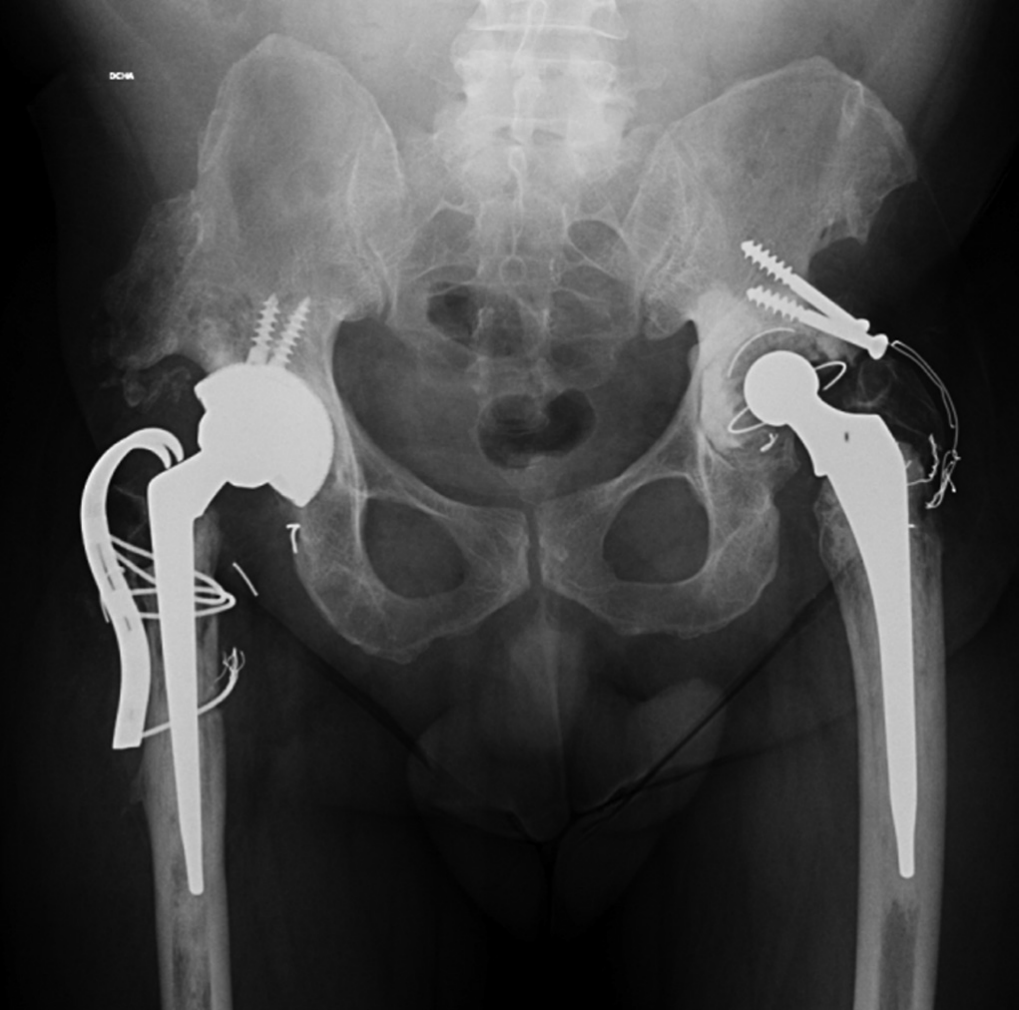
Anteroposterior radiograph of the pelvis where eccentric placement of the metal head can be seen. Signs of failure of the greater trochanter synthesis were also present.

For almost 2 years, the patient’s physical and radiological examinations were normal (Fig. 4a and b), showing nearly complete range of motion (10° of flexion deficit) and 5° of internal rotation. After that period, however, the patient began to feel progressive anterior groin pain and gait changes. An anteroposterior radiograph showed eccentric placement of the metal head and signs of failure in the greater trochanter synthesis (Fig. 5).

**Figure 6:**
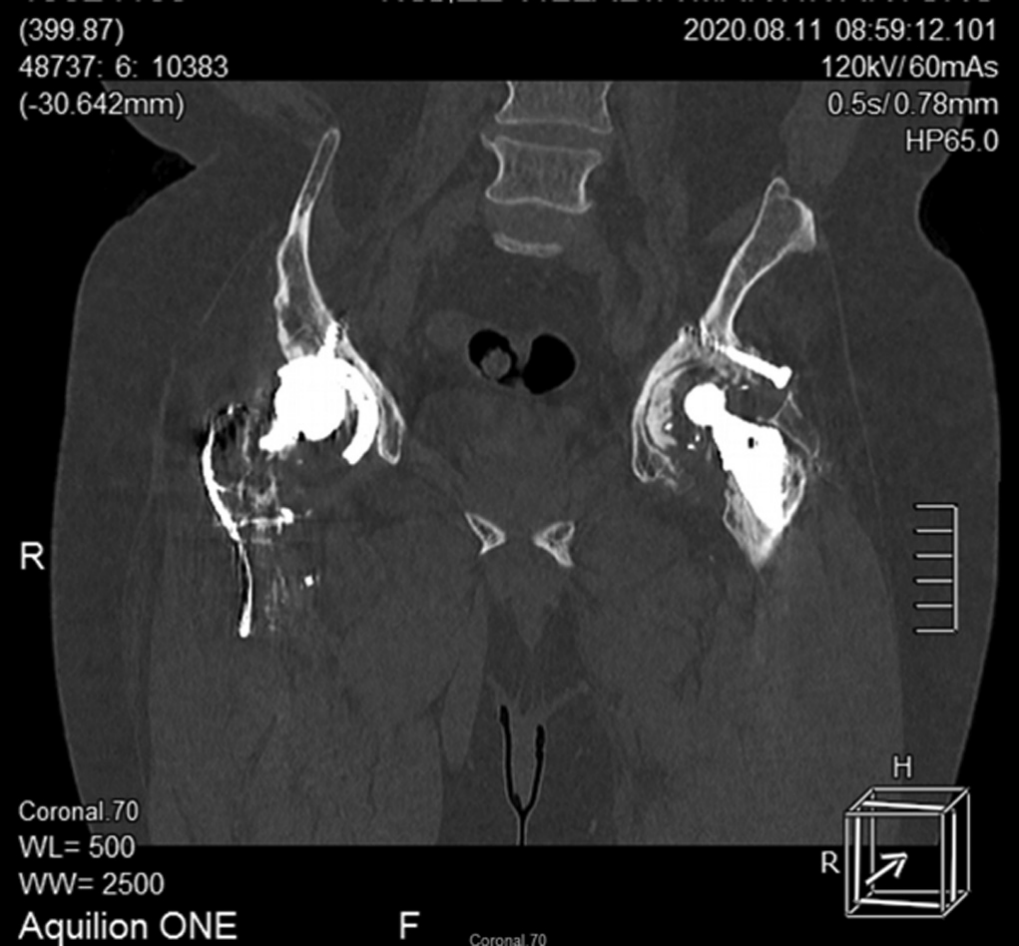
Coronal computed tomography image showing eccentricity of the metal head. Anteversion was reported to be 25°, and inclination was measured at 48°.

Although a computed tomography scan was prescribed, we lost contact with the patient for some time, and the scan was not performed until 4 months later. Eccentricity of the metal head was observed, but no circular radiolucent zone (“bubble sign” [6,20,23]) was present (Fig. 6). There was no evidence of elevated serum metal levels detected during his workup.

**Figure 7:**
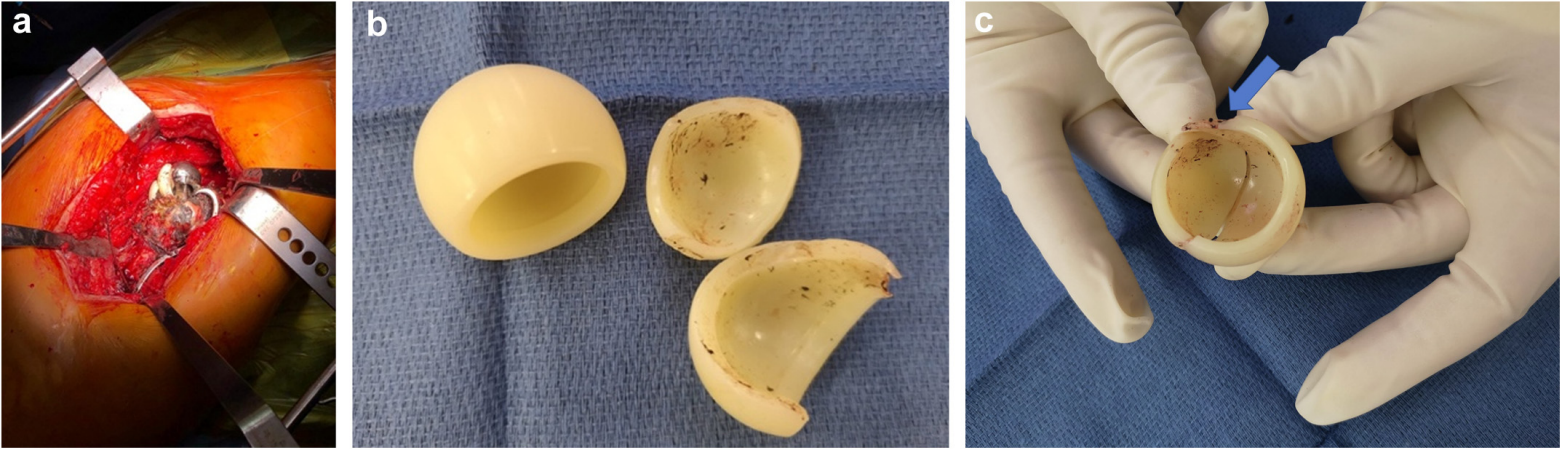
Intraoperative picture (a) and images of the broken insert (b and c) with a notched zone (arrow).

A second revision was performed, in which the polyethylene bearing was split into 2 parts with a notched area at the edge of the crack. No fragments of wire were found between the head and the insert or between the insert and the metal shell. The stem was stable, and the nonunion at the greater trochanter was still present (Fig. 7a-c).

**Figure 8:**
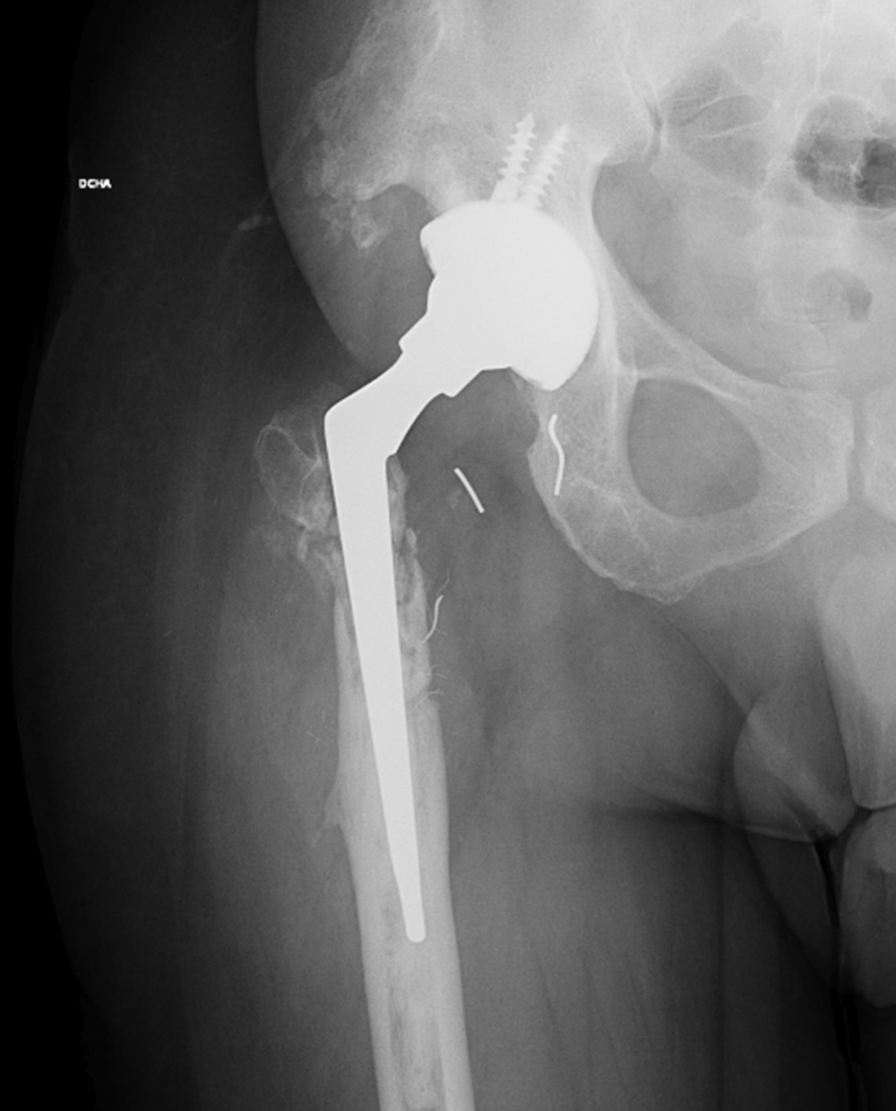
Postoperative anteroposterior radiograph of the hip after the second revision.

The integrity of the trunnion and the stability of both the cup and the dual-mobility liner were assessed intraoperatively. As no abnormalities were detected, the femoral head and the broken insert were removed and replaced with new ones. The well-fixed stem was left in place, and a long-neck skirted head (+10.5 mm) was chosen to provide adequate stability. The trochanteric plate was removed along with all visible and reachable wires, and the remaining bone of the trochanter was fixed to the femur by means of a tension band (FiberTape, Arthrex, FL). No complications were registered during or after the surgery (Fig. 8).

Postoperatively, the patient was able to ambulate without pain, and he was discharged from the hospital on day 5. One year after surgery, he had no further episodes of instability, pain, or gait alterations. To date, radiological examinations have shown no further changes.

An investigation report by the implant manufacturer stated that a visual examination of the components revealed normal signs of usage in the taper of the head and a fractured dual-mobility polyethylene bearing. The manufacturer could not identify the root cause of this issue and could not find any other comparable report.

## Discussion

We report a previously undescribed severe failure of a current DMC design in a symptomatic patient.

With the increasing use of DMCs, even in primary surgery and younger populations [3,25,26], concerns have arisen regarding future complications. Several authors have described potential issues related to DMC, with the most specific being IPD [4,6,[13], [14], [15], [16], [17]]. Several implant-related factors have been identified as possible causes of IPD, such as large-diameter bearings, use of bone cement, large-diameter femoral necks, small head-to-neck ratios, and long necks that include skirting [2,3,5,15,24,25]. The only proven patient-related cause is a high body mass index [15].

IPD is a rare complication, with reported incidence rates ranging from 1.9% to 5.2% with older-generation dual-mobility designs and from 0% to 2.4% with current designs [5]. The latest study of contemporary DMCs (implanted after 2000) reported no IPD at all [15]. This reduction can be explained because the new designs have addressed most of the mentioned causative factors [3]. Thus, the only head-to-bearing dislocations being reported in the literature are due to traumatic events [15,[18], [19], [20], [21], [22]].

However, our case does not fit in either of those categories. Although it might seem that the crack was caused during luxation or reduction, the patient had good function and was free of pain for 2 years after the traumatic episode. In addition, we could not find any other case where the implant split in such a drastic fashion. Owing to this lack of precedent, we hypothesize that this complication may have been caused by the following reasons:

− A notch, similar to what was described by Di Laura et al. [27], may have been caused by luxation (or reduction) and later evolved into a complete breakage of the polyethylene. The breakage of polyethylene liners in conventional THA is a rare but known complication [28] that has been related to the progression of fatigue cracks in modern, highly cross-linked polyethylene [29]. As a notch was present in the removed implant (Fig. 7C), we believe this is the most likely cause of the failure. That notch might have been caused by the trochanteric plate during the luxation or the reduction. Although we could not see impingement in different radiological projections, plate involvement cannot be ruled out.
− A crack may have occurred during the introduction of the snap-fit head and later progressed [30].
− Wires, bone cement debris or both may have caused third-body wear. However, we could not find any visible debris during the revision procedure.
− Fretting corrosion and metal ion release can generate an adverse local tissue reaction and all its dire consequences [16,31]. We left the stem of the original implant in place, which could be considered an “off-label” practice that could generate trunnionosis. We opted to leave the well-fixed cup and stem in place as proposed by other authors in an IPD without metallosis [6,16]. In our case, serum metal ion levels were normal, and we could not find trunnionosis or any adverse local tissue reaction.

We acknowledge that this study is limited in that it presents an isolated in vivo case of failure with no clear etiology. We are also aware that our management could raise several concerns. First, the patient’s attitude delaying the computed tomography scan could have increased the risk of metallosis [16,30,31]. Second, not replacing the stem and cup could be considered “off-label” use (mainly because the stem design plays a role in DMC [17]). Finally, we used a long-neck skirted bearing in the latest revision to add stability and to avoid the possible consequences of leaving the well-fixed stem in place. Although some of those decisions were debatable, we chose to avoid such an aggressive surgical step by not replacing the stem.

There is controversy surrounding the use of DMC. Although some authors show reluctance [5,31], many others encourage surgeons to consider DMC as a valid option for a wider range of patients, including young and active patients [3,15]. This duality could have its origin in the different clinical results obtained with the different implant generations. Current DMCs have better cup coatings, retaining mechanisms, materials, and designs than their predecessors. Nevertheless, as fixed-bearing articulations generally work well in high-demand young and active individuals, evidence of durability in this challenging population may be warranted before dual mobility is widely recommended for these patients. Of nearly 500 surgeries using DMC at our institution, this is the only case where we had such a failure, so we truly believe that DMC is a reliable solution that offers good stability and a broad range of movement in both primary and revision hip surgeries.

## Conflicts of interest

The authors declare that they have no known competing financial interests or personal relationships that could have appeared to influence the work reported in this article.

## Informed patient consent

The author(s) confirm that informed consent has been obtained from the involved patient(s) or if appropriate from the parent, guardian, power of attorney of the involved patient(s); and, they have given approval for this information to be published in this case report (series).

## Appendix A Supplementary data

Conflict of Interest Statement for Rodríguez Pérez

Conflict of Interest Statement for del Cármen Rodríguez

Conflict of Interest Statement for Tramunt Monsonet

Conflict of Interest Statement for Agulló Ferre
